# Comparison of decline in different cognitive domain in patients with normal pressure hydrocephalus

**DOI:** 10.1007/s10143-024-02410-3

**Published:** 2024-04-17

**Authors:** Ondřej Rýdlo, Adéla Bubeníková, Klára Häcklová, Petr Skalický, Róbert Leško, Adéla Ebelová, David Netuka, Vladimír Beneš IIIrd, Vladimír Beneš, Ondřej Bradáč

**Affiliations:** 1 Department of Neuropsychology, Second Medical Faculty, Charles University and Motol University Hospital, Prague, Czech Republic; 2 Department of Neurosurgery, Second Medical Faculty, Charles University and Motol University Hospital, Prague, Czech Republic; 3 Department of Neurosurgery and Neurooncology, First Medical Faculty, Charles University and Military University Hospital, Prague, Czech Republic; 4 Department of Neuropsychology, First Medical Faculty, Charles University and Military University Hospital, Prague, Czech Republic; 5 Department of Anaesthesiology and Intensive Care, Sligo University Hospital, Sligo, Ireland

**Keywords:** Normal pressure hydrocephalus, Neuropsychology, Cognitive decline, executive functions

## Abstract

We sought to describe the cognitive profile of patients with Idiopathic Normal Pressure Hydrocephalus (iNPH) using a comprehensive neuropsychological battery. Based on age and education correlated norms, we aimed to compare performance in each measured cognitive domain: executive functions (EFs), verbal memory (VM), non-verbal memory (nVM), visuoconstructional abilities (VA) and attention/psychomotor speed (A/PS). Patients diagnosed with iNPH underwent comprehensive neuropsychological evaluation before shunting. Their performance was compared to the age and education correlated norms. Correlation of different cognitive domains in iNPH profile was performed. A total of 53 iNPH patients (73.21 ± 5.48 years) were included in the study. All of the measured cognitive domains were significantly damaged. The most affected domains were EFs and VM (*p*<0.001 and *p*<0.001, respectively). A/PS domain was affected milder than EFs and VM (*p*<0.001). The least affected domains were nVM (*p*<0.001) and VA (*p*<0.001). Patients with iNPH are affected in all cognitive domains and the cognitive decline is uneven across these domains. The impairment of memory was shown to depend on the presented material. VM was shown to be much more severely affected than nVM and along with VM, EFs were shown to be the most affected. A/PS speed was shown to be less affected than VM and EFs and the least affected domains were nVM and VA.

**Supplementary Information:**

The online version contains supplementary material available at 10.1007/s10143-024-02410-3.

## Introduction

Idiopathic normal pressure hydrocephalus (iNPH) is a progressive syndrome manifested usually by the Hakim's triad: incontinence, gait disturbance and cognitive decline [1]. Gait disturbance is considered a primary symptom of iNPH and is present in 94–100% of patients. Cognitive decline is considered to be the second most prominent symptom reaching 78–98% frequency of onset [1].

In 1965, Adams et al. [2] described widening of lateral ventricles while maintaining unelevated intracranial pressure as a result of flow malfunction in cerebrospinal fluid (CSF). Hypoperfusion, glymphatic impairment, disturbance of metabolism, astrogliosis, neuroinflammation and blood-brain barrier disruption are speculated to cause white matter and gray matter lesions leading to typical iNPH symptoms [3]. The prevalence of iNPH is still discussed as there is a lack of homogeneity across the prevalence studies [4]. However, the recent review estimated prevalence ranging from 10/100 000 to 22/100 000 for probable iNPH and 29/100 000 for possible iNPH in the elderly population, increasing with higher age [4–7].

Despite the importance of cognitive decline in a manifestation of iNPH, a more detailed description is only now starting to be researched. Generally, cognitive deficits in iNPH patients are described as frontal and subcortical syndrome [5]. Symptoms of frontal impairment include slowing of psychomotor speed, deficits in attention, working memory and executive functions [8]. Subcortical dementias include a very similar profile and are characterized in addition by visuoconstructional deficits and memory impairment that manifests itself in the domain of retrieval rather than learning. This is often referred to as a fronto-subcortical deficit. Compared to cortical dementias, subcortical dementias are characterized by the absence of aphasia, apraxia and agnosia. Furthermore, they are characterized by a non-hippocampal type of memory deficit and preserved awareness of their illness [9]. Of note is that the division of subcortical and cortical impairments is not universally accepted since the two types considerably overlap each other [10, 11].

One of the most affected cognitive areas in patients with iNPH is executive function [12–14]. Executive functions contribute to planning, decision-making, judgment, inhibiting actions and other mental systems that lead to purposeful behavior and its regulation [9]. Furthermore, psychomotor pace is usually slowed in iNPH patients [15]. Psychomotor pace refers to the ability to perceive and respond to changes in the environment, such as the presentation of a specific stimulus [16]. Psychomotor pace can also be clinically assessed as the ability to respond verbally or motorically at an appropriate speed to a given situation. Some authors define psychomotor pace more broadly as the processing speed at which the brain is capable of working [17]. Although normal pressure hydrocephalus is not a typical cortical dementia like Alzheimer's disease, it is shown to also affect the patient's memory functions, however, it seems to affect retrieval rather than storing process of memory. Additionally, visuoconstructional abilities seem to be disrupted in patients with iNPH, especially when assessed by more complex figures [18]. This article aims to evaluate characteristics of the neuropsychological cognitive profile in iNPH patients and neuropsychological interdomain correlations.

## Materials and methods

### Participants

A total of 126 patients withsuspected iNPH were examined at the Department of Neurosurgery and Neuro-oncology between 2018 and 2021. After admission, patients underwent a complex diagnostic protocol consisting of clinical, psychological, imaging and functional examinations. In order to have been included in the study, all patients had to have had (1) onset of symptoms over period of at least 3 months, (2) Evans’ index > 0.3, (3) gait disturbance and at least one of the remaining Hakim’s triad symptoms, (4) no other known underlying condition that would account for the symptoms. Charlson’s comorbidity index [19] was used to assess the patient’s overall prognosis in further detail.

Patients fulfilling these criteria underwent lumbar infusion test (LIT). Once the test was completed, lumbar drainage was performed using the same needle, CSF was drained for 120 hours and clinical evaluation was repeated. All patients with at least a 15% improvement on the Dutch Gait Scale [20] clinical and imaging criteria, were evaluated as probable iNPH and underwent VP shunt implantation. If these criteria were not fulfilled, the patient was determined as non-iNPH, referred for further outpatient surveillance. Patients with abnormal CSF opening pressure (> 20 cm H_2_O) and/or abnormal laboratory findings in CSF were excluded from the study. The psychologist was not informed of either MRI or functional tests evaluations. The neuropsychological assessment took place within the standard clinical setting, where a trained neuropsychologist conducted the neuropsychological battery with the patient during one session prior to VP shunt implantation.

### Neuropsychological battery

The neuropsychological battery consisted of the Montreal Cognitive Assessment [21, 22], which includes subtests assessing the patient's visuoconstructional, executive functions, memory, attention, and verbal functions. Additionally, the Trail Making Test in both Form A and Form B (TMT A, B) [23, 24], as well as Auditory Verbal Learning Test (AVLT) [25, 26] were administered. We measured time of completion for TMT A, B and number of remembered words in AVLT. Furthermore, the Rey-Osterrieth Complex Figure Test (ROCFT) [27] was included in the protocol, consisting of copy, reproduction (3 min after copy) and delayed recall (30 min after copy). The trained neuropsychologist evaluated each drawing using Drozd's criteria [28], which assess the figure based on placement and accuracy of each individual segment. From the Wechsler tests, the Block Design subtest [29, 30] was administered, where the patient is required to assemble white and red blocks to match a given design. Raw score yielded according to the test manual is used for statistical analysis. Finally, two verbal fluency tests were administered – Letter Fluency (czech standardized version of FAS test for letters N, K and P) and Category Fluency (Animal and Vegetable) [9, 31]. The total amount of provided words for 1 minute was the final indicator for analysis. Details on manuals and normative data of neuropsychological tests used in this study are presented in the Supplementary Material.

**Fig. 1:**
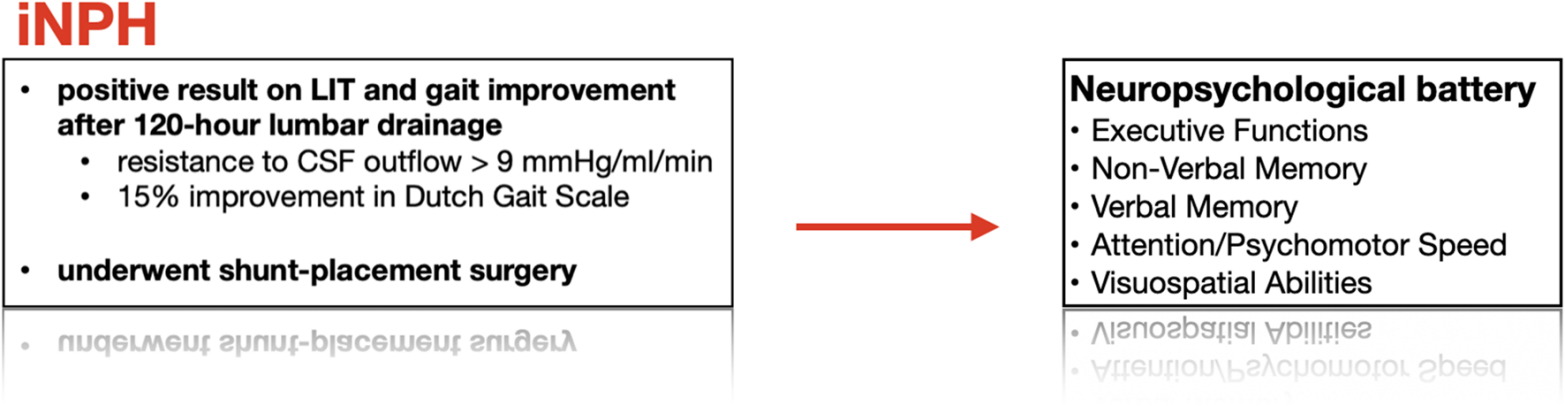
Diagram depicting the patient selection in the present study. Used abbreviations: CSF, cerebrospinal fluid; LIT, lumbar infusion test. The Dutch Gait Scale includes a test of 10 features of walking and the number of steps and seconds necessary for 10 m

As indicators of executive functioning, the TMT-B, Block Design test, letter and category fluency tests were analyzed. Memory abilities were divided into verbal and non-verbal components. The verbal component was assessed through the results of the AVLT, Letter Fluency, and Category Fluency tests, while the non-verbal component was evaluated using the ROCFT – reproduction (3 min) and delayed recall (30 min). For the verbal part, we excluded all patients who did not complete AVLT, as it is the most important test in this category. Visuoconstructional abilities were measured using the ROCFT-copy and Block Design tests. Attention/psychomotor pace were assessed using the TMT-A and ROCFT-copy tests. For the analysis of each domain, we excluded all patients who did not complete at least two tests in the specific domain. The process of patient selection is depicted in Fig. 1.

### Statistical analysis

The results of neuropsychological tests were transformed to t-scores according to individual manuals with respect to age and education correlated norms. The normality of the data was assessed according to the Kolmogorov-Smirnov test. We deployed one sample t-test to reveal the differences of iNPH patients against the average of normal population using standard metrics of t-statistic (*m* = 50, sd = 10). For the correlation among the individual cognitive domains, Pearson's coefficient was used. All statistical analyses were conducted with Jamovi statistical software (v2.3, The Jamovi Project 2022, https://www.jamovi.org/). Graphical interpretations were modeled using Hmisc and Corrplot libraries in the open-source R environment (v4.1.2, R Core Team (2021). Vienna, Austria, https://www.R-project.org/).

## Results

The resulting sample consisted of 53 iNPH patients who were indicated for VP shunt implantation and underwent neuropsychological evaluations with the mean age of 73.21 ± 5.48 years, 64% (*n*=34) were men. The mean pre-LD in Dutch Gait Scale was 26.4 ± 9.9, while post-ELD Dutch Gait Score was 18.8 ± 10.8 (*p*<0.001). Moreover, the median Charlson’s comorbidity index was 6 points. All patients had at least two symptoms from the Hakim’s triad. The majority of patients harbored all three (77.7%). For the analysis, we attempted to use all the iNPH confirmed patients, however, not all of them accomplished all the required tests embraced in the neuropsychological battery. Hence, for the analysis of executive functions, we used data of 38 patients (36.6 ± 5.15); 37 patients (39.5 ± 10.12) for the analysis of non-verbal memory; 37 patients (34.1 ± 4.72) for the analysis of verbal memory; 40 patients (37.4 ± 8.14) for the analysis of attention/psychomotor speed. For the analysis of visuoconstruction, we used data of 38 patients (41.3 ± 8.72). The description of each sample for the analysis can be found in Table 1.

**Table 1:** Descriptive statistics of included patients in this study with provided characteristics of t-tests among studied cognitive domains. Used abbreviations: SD, standard deviation; SE, standard error

	N. of patients	Mean t-score	SD	SE
Executive Functions	38	36.6	5.15	0.836
Non-Verbal Memory	37	39.5	10.12	1.664
Verbal Memory	37	34.1	4.72	0.775
Attention/Psychomotor Speed	40	37.4	8.14	1.287
Visuoconstructional Abilities	38	41.3	8.72	1.414

**Fig. 2:**
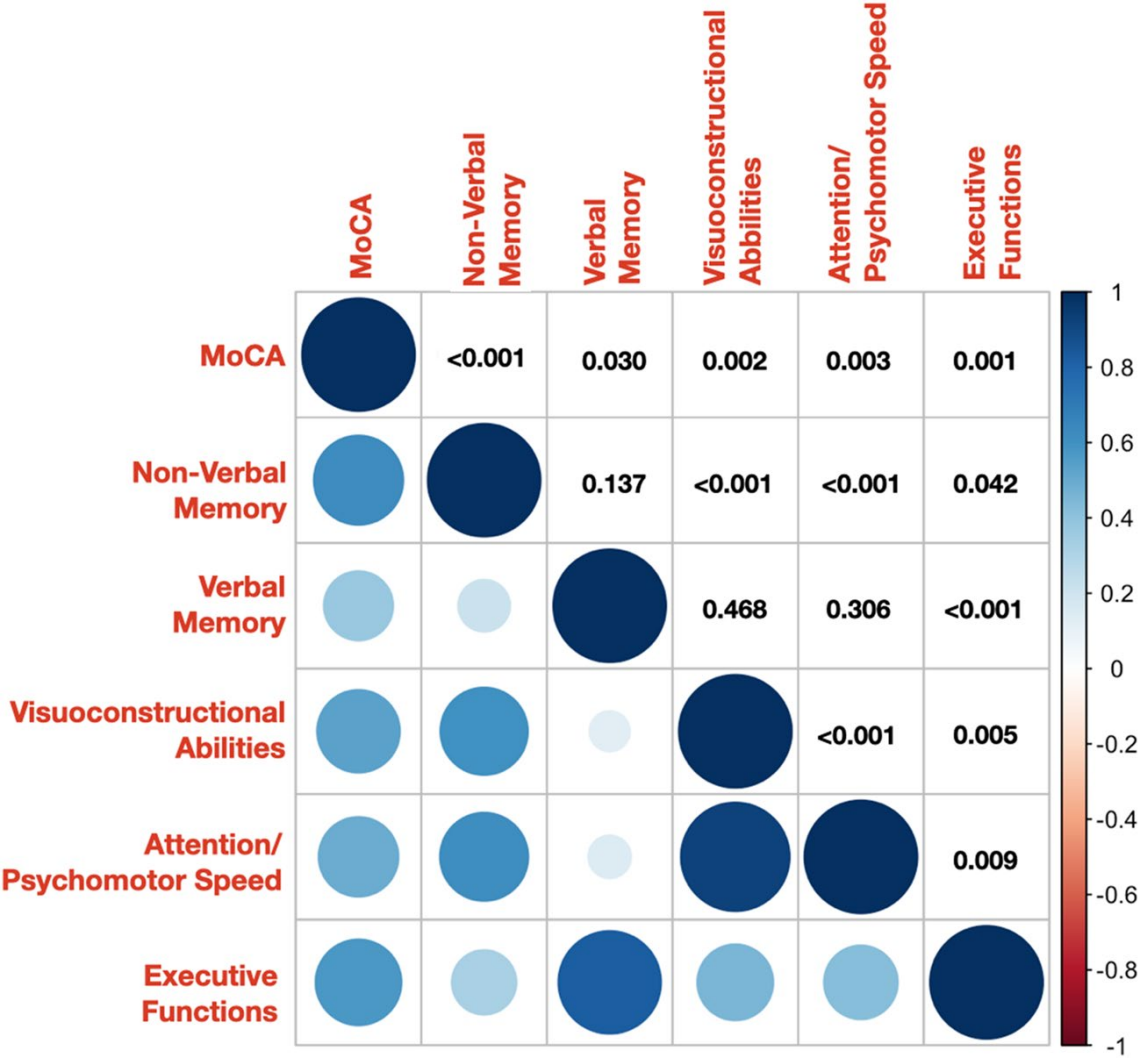
Correlation matrix of neuropsychological interdomain relationships. Presented numbers are p-values of each individual interdomain correlations. Used abbreviations: MoCA, Montreal Cognitive Assessment

We found strong correlation between executive functions and verbal memory (*r*=0.829; *p*<0.001) or attention and visuoconstructional abilities (*r*=0.917; *p*<0.001). Moderate correlation was found between all neuropsychological domains and MoCA test, suggesting that cognitive decline progresses gradually across the domains. See Figure 2 for more details.

We found significant results for all of the observed cognitive domains with iNPH group scoring significantly lower (see Table 2). A statistically significant result was found for executive functions domain (*t*=-16.01, *p*<0.001) with a very large effect size (*d*=-2.6). Analogous results were obtained for (1) the verbal memory (*t*=-20.48, *p*<0.001, *d*=-3.36); (2) the visuoconstruction (*t*=-6.16, *p*<0.001, *d*=-0.99); and (3) the non-verbal memory (*t*=55.5, *p*<0.001, *d*=-1.04). Finally, also for (4), the attention/psychomotor speed, we found a statistically significant difference (*t*=18, *p*<0.001) with a large effect size (*d*=-1.542).

**Table 2:** Results of statistical tests in all measured cognitive domains in patients with iNPH. All effect sizes were measured using Cohen’s d statistics

Domain	*p*-value	Effect Size
Executive Functions	< 0.001	-2.597
Verbal Memory	< 0.001	-3.366
Visuoconstructional abilities	< 0.001	-0.999
Non-Verbal Memory	< 0.001	-1.04
Attention/Psychomotor Speed	< 0.001	-1.542

## Discussion

Our results confirm that iNPH patients are severely affected in all cognitive domains measured. Moreover, we found that verbal memory and executive functions are one of the most affected cognitive domains in this diagnosis. These findings concur with previous research [12, 20]. We found attention/psychomotor speed to be less impaired than executive functions and verbal memory. Additionally, we found non-verbal memory and visuoconstructional abilities to be the least impaired of the measured cognitive domains in iNPH patients. However, it is important to note that all of the measured cognitive domains were considered in regard to educational and age correlated norms and all of them were severely impaired in that respect.

As pointed out in the introduction of this article, all of these cognitive domains are associated with the iNPH diagnosis. There is no common understanding however, of how much the individual domains are impaired when viewed in relation to each other. Thus, we offer our results as an invitation to further scrutiny. We found that verbal memory is much more impaired than non-verbal. These results suggest that iNPH damages memory unevenly. It is an interesting result, nevertheless, it is important to note that we measured non-verbal memory with only one neuropsychological test (ROCFT), and thus we recommend using more neuropsychological tests in the future which measure non-verbal memory in more detail, such as for example Brief Visual Memory Test - Revised. Also, the results of verbal memory in our observed group relies heavily on AVLT, which is a difficult task to perform. Considering the progress of cognitive decline, it can be suggested that in later stages the AVLT test has a smaller differentiating ability and may thus lead to potentially biased results as the floor effect could slightly skew the results. On the other hand, we used Fonemic and Categorical Verbal Fluency tests to assess the same cognitive domain – verbal memory in iNPH patients, which should improve the results and provide a more comprehensive picture of iNPH patient’s memory. The strong correlation appeared between executive functioning and verbal memory, while not between executive functioning and non-verbal memory supporting the idea that memory is impaired unevenly based on the presented material.

Our results additionally confirmed that executive functions are severely impaired in iNPH. This is in accordance with previous research [12, 13, 18]. It seems that inhibition and shifting ability of executive functions are impaired [12, 32, 33], however data on ability to maintain the activity are missing and the data on updating ability measured by Digit span backward test are mixed, impaired [32] and not significant [34]. Detailed description of executive functioning might help better diagnostic quality of cognitive tests in the future.

From our study, it seems that visuoconstructional abilities are the least affected cognitive domain in iNPH patients, even though it is still impaired compared to healthy individuals. Possible explanation might be that visuoconstructional abilities are affected in the later stages of the illness and hence are less severely impaired in general. It might be interesting to observe the progress of visuoconstructional abilities in iNPH with respect to its potential diagnostic value. Lastly, we did not focus on language abilities in this study, however, from the previous research it is possible to assume that verbal fluency is impaired but naming ability mostly remains intact [15].

Compared to Alzheimer's disease (AD), iNPH cognitive profile is characterized by frontal lobe symptoms and disproportionately impaired memory function [8]. Spontaneous recall seems to be impaired in both, AD [18, 35] and iNPH, however, in iNPH the role of clue is crucial and should normalize the memory performance as it is theorized as subcortical type of cognitive decline [5]. It might be due to a fact that iNPH does not typically affect the hippocampus as AD does [36]. Saito et al. [18] highlight the episodic part of memory to be more impaired in AD than in iNPH and also depict visuoconstructional abilities as more damaged in iNPH than AD. This finding is supported by Nerg et al. (2021) [37]. They used a comprehensive CERAD Neuropsychological Battery in iNPH, AD and healthy patients and concluded that 1) iNPH are more affected on all performed tests than healthy patients; 2) iNPH performed worse on verbal fluency (which might reflect either slower psychomotor pace or impaired semantic fluency) and Clock Drawing test (which might reflect worse visuoconstructional abilities); 3) AD patients had more severe episodic memory dysfunction than iNPH (and healthy patients). Moreover, attentional functions seem to be less severely impaired in AD patients rather than memory [38], unlike in iNPH where the attention along with psychomotor pace is theorized to be impaired quite early in the process [15]. On the other hand, in Frontotemporal Lobar Degeneration (FTLD), the cognitive decline is manifested by the impairment in executive functions and slower psychomotor speed much like we observe in iNPH [39]. Nevertheless, for example Diehl and Kurz [38] found in their study that patients with frontotemporal dementia performed better in visuoconstructional abilities not only in comparison to AD group but also performed equally in comparison to healthy control group. This would suggest no visuoconstructional impairment in Frontotemporal Dementia (FTD) and might be a differentiating factor between FTD and iNPH. Also, along with FTD and especially its most common subgroup behavioral variant FTD, there are often psychiatric symptoms and frequent changes in personalities [40, 41]. This is not a well studied topic in iNPH and might be a very interesting field to research in the future. Lastly, compared to Parkinson's disease (PD), iNPH cognitive disabilities arise earlier in the stages of the illness and with more diffuse impairment. Only 25% of PD showed executive dysfunctions compared to 65 % of iNPH [42].

## Study strengths and limitations

One of the limitations is the comparison of iNPH patients to standardized norms from different neuropsychological tests rather than healthy adult control groups. However, comparison of different cognitive domains in this study might serve as a solid ground to build a profound cognitive profile of iNPH patients. Secondarily, we did not discriminate between different stages of the illness in our cohort, thus reflecting one cognitive profile instead of detailed description of the development of overall iNPH cognitive decline. Also, the results might be skewed due to lack of differentiation between progression of the illness as the cognitive decline in later stages is global and cannot be easily differentiated from other cognitive declines such as the one with AD or PD [15]. Some cognitive domains were not covered with more neuropsychological tests and it might be worth examining them in more depth in the future. Lastly, the interpretation of A/PS domain is limited, as we used ROCFT for assessing this domain, which is not traditional, but it has been assessed in a recent publication [27]. We believe that it can provide useful insight for further scrutiny.

## Conclusion

Patients with iNPH are affected in all cognitive domains and the cognitive decline is uneven across these domains. The impairment of memory depends on the presented material. Verbal memory seems to be much more severely affected than non-verbal memory. Along with VM, the EFs are the most affected. Attention/Psychomotor speed is less affected than VM and EFs. The least affected domains are nVM and VA. However, describing the progress of cognitive decline in more detail might be fruitful in future. Nevertheless, overall our study might be used for researchers to focus cognitive functioning in iNPH in a more precise way and for practitioners, it might serve as a cornerstone for deciding which neuropsychological tests to deploy.

## Supplementary information

ESM 1 (PDF 64 kb)

## References

[1] Nakajima M, Yamada S, Miyajima M, Ishii K, Kuriyama N, Kazui H, Kanemoto H, Suehiro T, Yoshiyama K, Kameda M et al (2021) Guidelines for management of idiopathic normal pressure hydrocephalus (third edition): endorsed by the Japanese society of normal pressure hydrocephalus, Neurol Med Chir (Tokyo) 61(63–97). 10.2176/nmc.st.2020-0292 10.2176/nmc.st.2020-0292 PMC7905302 33455998

[2] Adams RD, Fisher CM, Hakim S, Ojemann RG, Sweet WH (1965) Symptomatic occult hydrocephalus with "normal" cerebrospinal-fluid pressure.a treatable syndrome. N Engl J Med 273:117–126. 10.1016/10.1056/nejm196507152730301 14303656 10.1056/NEJM196507152730301

[3] Wang Z, Zhang Y, Hu F, Ding J, Wang X (2020) Pathogenesis and pathophysiology of idiopathic normal pressure hydrocephalus. CNS Neurosci Therapeut 26:1230–1240. 10.1111/cns.13526 10.1111/cns.13526 PMC7702234 33242372

[4] Zaccaria V, Bacigalupo I, Gervasi G et al (2020) A systematic review on the epidemiology of normal pressure hydrocephalus. Acta Neurol Scand 141:101–114. 10.1111/ane.13182 31622497 10.1111/ane.13182

[5] Shprecher D, Schwalb J, Kurlan R (2008) Normal pressure hydrocephalus: diagnosis and treatment. Curr Neurol Neurosci Rep 8:371–376. 10.1007/s11910-008-0058-2 18713572 10.1007/s11910-008-0058-2 PMC2674287

[6] Andersson J, Rosell M, Kockum K, Lilja-Lund O, Söderström L, Laurell K (2019) Prevalence of idiopathic normal pressure hydrocephalus: A prospective, population-based study. Plos One 14:e0217705. 10.1371/journal.pone.0217705 31141553 10.1371/journal.pone.0217705 PMC6541279

[7] Tanaka N, Yamaguchi S, Ishikawa H, Ishii H, Meguro K (2008) Prevalence of Possible Idiopathic Normal-Pressure Hydrocephalus in Japan: The Osaki-Tajiri Project. Neuroepidemiology 32:171–175. 10.1159/000186501 19096225 10.1159/000186501

[8] Ogino A, Kazui H, Miyoshi N, Hashimoto M, Ohkawa S, Tokunaga H, Ikejiri Y, Takeda M (2006) Cognitive impairment in patients with idiopathic normal pressure hydrocephalus. Dement Geriatr Cogn Disord 21(113–119). 10.1159/000090510 10.1159/000090510 16374006

[9] Kapur N (2005) Neuropsychological Assessment, Fourth Edition. J Neurol 252:1290–1291. 10.1007/s00415-005-0003-0

[10] Turner MA, Moran NF, Kopelman MD (2002) Subcortical dementia. The British J Psychiatr 180:148–151. 10.1192/bjp.180.2.148 10.1192/bjp.180.2.148 11823326

[11] Brown RG, Marsden CD (1988) 'Subcortical dementia': the neuropsychological evidence. Neuroscience 25:363–387. 10.1016/0306-4522(88)90246-1 2969464 10.1016/0306-4522(88)90246-1

[12] Bugalho P, Alves L, Miguel R, Ribeiro O (2014) Profile of cognitive dysfunction and relation with gait disturbance in Normal Pressure Hydrocephalus. Clin Neurol Neurosurg 118:83–88. 10.1016/j.clineuro.2014.01.006 24529236 10.1016/j.clineuro.2014.01.006

[13] Devito EE, Pickard JD, Salmond CH, Iddon JL, Loveday C, Sahakian BJ (2005) The neuropsychology of normal pressure hydrocephalus (NPH). British Journal of Neurosurgery 19(217–224). 10.1080/02688690500201838 10.1080/02688690500201838 16455521

[14] Solana E, Sahuquillo J, Junqué C, Quintana M, Poca MA (2012) Cognitive Disturbances and Neuropsychological Changes after Surgical Treatment in a Cohort of 185 Patients with Idiopathic Normal Pressure Hydrocephalus. Arch Clin Neuropsychol 27:304–317. 10.1093/arclin/acs002 22382387 10.1093/arclin/acs002

[15] Xiao H, Hu F, Ding J, Ye Z (2022) Cognitive Impairment in Idiopathic Normal Pressure Hydrocephalus. Neurosci Bull 38:1085–1096. 10.1007/s12264-022-00873-2 35569106 10.1007/s12264-022-00873-2 PMC9468191

[16] Carone DA, Strauss E, Sherman EMS, Spreen O (2007) A compendium of neuropsychological tests: administration, norms, and commentary. Appl Neuropsychol 14:62–63. 10.1080/09084280701280502

[17] Keys BA, White DA (2000) Exploring the relationship between age, executive abilities, and psychomotor speed. J Int Neuropsychol Soc 6:76–82. 10.1017/S1355617700611098 10761370 10.1017/s1355617700611098

[18] Saito M, Nishio Y, Kanno S, Uchiyama M, Hayashi A, Takagi M, Kikuchi H, Yamasaki H, Shimomura T, Iizuka O et al (2011) Cognitive Profile of Idiopathic Normal Pressure Hydrocephalus. Dement Geriatr Cogn Disord Extra 1:202–211. 10.1159/000328924 10.1159/000328924 PMC3199897 22163245

[19] Charlson ME, Pompei P, Ales KL, MacKenzie CR (1987) A new method of classifying prognostic comorbidity in longitudinal studies: development and validation. J Chronic Dis 40(5):373–383. 10.1016/0021-9681(87)90171-8 3558716 10.1016/0021-9681(87)90171-8

[20] Boon AJ, Tans JT, Delwel EJ, Egeler-Peerdeman SM, Hanlo PW, Wurzer HA, Hermans J (2000) The Dutch normal-pressure hydrocephalus study. How to select patients for shunting? An analysis of four diagnostic criteria. Surg Neurol 53:201–207. 10.1016/s0090-3019(00)00182-8 10773249 10.1016/s0090-3019(00)00182-8

[21] Nasreddine ZS, Phillips NA, Bédirian V, Charbonneau S, Whitehead V, Collin I, Cummings JL, Chertkow H (2005) The Montreal Cognitive Assessment, MoCA: A Brief Screening Tool For Mild Cognitive Impairment. J Am Geriatr Soc 53:695–699. 10.1111/j.1532-5415.2005.53221.x 15817019 10.1111/j.1532-5415.2005.53221.x

[22] Kopecek M, Stepankova H, Lukavsky J, Ripova D, Nikolai T, Bezdicek O (2017) Montreal cognitive assessment (MoCA): Normative data for old and very old Czech adults. Appl Neuropsychol: Adult 24(1):23–29. 10.1080/23279095.2015.1065261 27144665 10.1080/23279095.2015.1065261

[23] Bowie CR, Harvey PD (2006) Administration and interpretation of the Trail Making Test. Nat Protoc 1:2277–2281. 10.1038/nprot.2006.390 17406468 10.1038/nprot.2006.390

[24] Bezdicek O, Motak L, Axelrod BN, Preiss M, Nikolai T, Vyhnalek M et al (2012) Czech version of the Trail Making Test: Normative data and clinical utility. Arch Clin Neuropsychol 27(8):906–914. 10.1093/arclin/acs084 23027441 10.1093/arclin/acs084

[25] Rey A (1964) L' examen clinique en psychologie, 2nd edn. Presses universitaires de France, Paris, France

[26] Bezdicek O, Stepankova H, Motak L, Axelrod BN, Woodard JL, Preiss M, Nikolai T, Růžička E, Poreh A (2014) Czech version of Rey Auditory Verbal Learning test: Normative data. Aging Neuropsychol Cogn 21(6):693–721. 10.1080/13825585.2013.865699 10.1080/13825585.2013.865699 24344673

[27] Zhang X, Lv L, Min G, Wang Q, Zhao Y, Li Y (2021) Overview of the complex figure test and its clinical application in neuropsychiatric disorders, including copying and recall. Front Neurol 12:680474. 10.3389/fneur.2021.680474 34531812 10.3389/fneur.2021.680474 PMC8438146

[28] Drozdova K, Stepankova Georgi H, Lukavsky J, Bezdicek O, Kopecek M (2015) Normative data for the reyosterrieth complex figure test in older Czech adults. Česká a Slovenská neurologie a neurochirurgie 78(111):542–549

[29] Kaufman AS (1983) Test Review: Wechsler, D. Manual for the Wechsler Adult Intelligence Scale, Revised. New York: Psychological Corporation, 1981. J Psychoeduc Assess 1:309–313. 10.1177/073428298300100310

[30] Černochová D, Goldmann P, Král P, Soukupová T, Šnorek P, Havlůj V (2010) Wechslerova inteligenční škála pro dospělé WAIS III. Praha. Hogrefe-Testcentrum, Czech republic

[31] Nikolai T, Štěpánková H, Michalec J, Bezdíček O, Horáková K, Marková H, Kopeček M (2015) Testy verbální fluence, česká normativní studie pro osoby vyššího věku. Česká a slovenská neurologie a neurochirurgie 78, 111(3):292–299

[32] da Rocha SFB, Kowacs PA, de Souza RKM, Pedro MKF, Ramina R, Teive HAG (2021) Serial Tap Test of patients with idiopathic normal pressure hydrocephalus: impact on cognitive function and its meaning. Fluids Barriers CNS 18:22. 10.1186/s12987-021-00254-3 33957939 10.1186/s12987-021-00254-3 PMC8101193

[33] Hülser M, Spielmann H, Oertel J, Sippl C (2022) Motor skills, cognitive impairment, and quality of life in normal pressure hydrocephalus: early effects of shunt placement. Acta Neurochirurg 164:1765–1775. 10.1007/s00701-022-05149-2 10.1007/s00701-022-05149-2 PMC9233626 35212797

[34] Gleichgerrcht E, Cervio A, Salvat J, Loffredo AR, Vita L, Roca M, Torralva T, Manes F (2009) Executive function improvement in normal pressure hydrocephalus following shunt surgery. Behav Neurol 21:181–185. 10.3233/BEN-2009-0249 19996515 10.3233/BEN-2009-0249 PMC5444271

[35] Sahakian BJ, Downes JJ, Eagger S, Everden JL, Levy R, Philpot MP, Roberts AC, Robbins TW (1990) Sparing of attentional relative to mnemonic function in a subgroup of patients with dementia of the Alzheimer type. Neuropsychologia 28:1197–1213. 10.1016/0028-3932(90)90055-S 2290494 10.1016/0028-3932(90)90055-s

[36] Vlasák A, Skalický P, Mládek A, Vrána J, Beneš V, Bradáč O (2021) Structural volumetry in NPH diagnostics and treatment—future or dead end? Neurosurg Rev 44:503–514. 10.1007/s10143-020-01245-y 31980974 10.1007/s10143-020-01245-y

[37] Nerg O, Junkkari A, Hallikainen I, Rauramaa T, Luikku A, Hiltunen M, Jääskeläinen JE, Leinonen V, Hänninen T, Koivisto A (2021) The CERAD Neuropsychological Battery in Patients with Idiopathic Normal Pressure Hydrocephalus Compared with Normal Population and Patients with Mild Alzheimer's Disease. J Alzheimers Dis 81(3):1117–1130. 10.3233/JAD-201363 33896842 10.3233/JAD-201363

[38] Diehl J, Kurz A (2002) Frontotemporal dementia: patient characteristics, cognition, and behaviour. Int J Geriatr Psychiatr 17:914–918. 10.1002/gps.709 10.1002/gps.709 12325050

[39] Korhonen VE, Solje E, Suhonen NM, Rauramaa T, Vanninen R, Remes AM, Leinonen V (2017) Frontotemporal dementia as a comorbidity to idiopathic normal pressure hydrocephalus (iNPH): a short review of literature and an unusual case. Fluids Barriers CNS 14(10). 10.1186/s12987-017-0060-7 10.1186/s12987-017-0060-7 PMC5395836 28420385

[40] Serggio CL, Bruce LM (2016) The behavioural variant frontotemporal dementia (bvFTD) syndrome in psychiatry. J Neurol Neurosurg Psychiatr 87:501. 10.1136/jnnp-2015-310697 10.1136/jnnp-2015-310697 PMC4755931 26216940

[41] Picascia M, Pozzi NG, Todisco M, Minafra B, Sinforiani E, Zangaglia R, Ceravolo R, Pacchetti C (2019) Cognitive disorders in normal pressure hydrocephalus with initial parkinsonism in comparison with de novo Parkinson's disease. Eur J Neurol 26:74–79. 10.1111/ene.13766 30091839 10.1111/ene.13766

[42] Golomb J, Wisoff J, Miller DC, Boksay I, Kluger A, Weiner H, Salton J, Graves W (2000) Alzheimer’s disease comorbidity in normal pressure hydrocephalus: prevalence and shunt response. J Neurol Neurosurg Psychiatr 68:778. 10.1136/jnnp.68.6.778 10.1136/jnnp.68.6.778 PMC1736969 10811706

