# Supplementary material for: Comparison of decline in different cognitive domain in patients with normal pressure hydrocephalus
Source: Neurosurg Rev. 2024 Apr 17;47(1):167. doi: 10.1007/s10143-024-02410-3 (PMC11024017; doi:10.1007/s10143-024-02410-3)
Supplement: Supplementary file 1 — (PDF 64 kb) [file 10143_2024_2410_MOESM1_ESM.pdf]

Used manuals for neuropsychological tests:

TMT-A	Bezdicek, O., Motak, L., Axelrod, B. N., Preiss, M., Nikolai, T., Vyhnalek, M., ... & Ruzicka, E. (2012). Czech version of the Trail Making Test: Normative data and clinical utility. *Archives of Clinical Neuropsychology*, *27*(8), 906-914.
TMT-B	Bezdicek, O., Motak, L., Axelrod, B. N., Preiss, M., Nikolai, T., Vyhnalek, M., ... & Ruzicka, E. (2012). Czech version of the Trail Making Test: Normative data and clinical utility. *Archives of Clinical Neuropsychology*, *27*(8), 906-914.
AVLT	Bezdicek, O., Stepankova, H., Motak, L., Bradley N. Axelrod, Woodard, John L., Preiss, M., Nikolai, T., Růžička, E. & Poreh A. (2014) Czech version of Rey Auditory Verbal Learning test: Normative data, Aging, Neuropsychology, and Cognition, 21:6, 693-721
ROCFT	Drozdova, K., Stepankova, H., Lukavsky, J., Bezdicek, O., & Kopecek, M. (2015). Normative data for the rey-osterrieth complex figure test in older Czech adults. *Ceska a Slovenska Neurologie a Neurochirurgie*, *78*(5), 542-549.
Block design	Černochová, D., Goldmann, P., Král, P., Soukupová, T., Šnorek, P., & Havlůj, V. (2010). Wechslerova inteligenční škála pro dospělé WAIS III. Praha. *Czech republic: Hogrefe-Testcentrum*.
Verbal Fluency	Nikolai, T., Štěpánková, H., Michalec, J., Bezdíček, O., Horáková, K., Marková, H., & Kopeček, M. (2015). Testy verbální fluence, česká normativní studie pro osoby vyššího věku. *Česká a slovenská neurologie a neurochirurgie, 78*, *111*(3), 292-299.
